# Geese Reared in Vineyard: Soil, Grass and Animals Interaction

**DOI:** 10.3390/ani9040179

**Published:** 2019-04-19

**Authors:** Luisa Massaccesi, Alice Cartoni Mancinelli, Simona Mattioli, Mauro De Feudis, Cesare Castellini, Alessandro Dal Bosco, Maria Laura Marongiu, Alberto Agnelli

**Affiliations:** 1 Department of Agricultural, Environmental and Food Science, University of Perugia, Borgo XX Giugno 74, 06124 Perugia, Italy; luisa.massaccesi@gmail.com (L.M.); simona.mattioli@hotmail.it (S.M.); maurodfagr@gmail.com (M.D.F.); cesare.castellini@unipg.it (C.C.); alessandro.dalbosco@unipg.it (A.D.B.); alberto.agnelli@unipg.it (A.A.); 2 Department of Veterinary Medicine, University of Sassari, Via Vienna 2, 07100 Sassari, Italy; marongiu@uniss.it

**Keywords:** geese, copper, vineyard soil properties, agroforestry

## Abstract

**Simple Summary:**

Agroforestry is a practice, which consists of having orchard, crops and animals in the same land. This system shows many sustainability advantages like reductions of land use, permitting to obtain two productions (vegetal and animal) in the same area. Moreover, if the animals are well managed, they exert a double action by fertilizing and weeding the soil. The agroforestry system here studied consists of an organic vineyard where geese at two densities (High Geese Density-HGD and Low Geese Density-LGD) were reared. In the organic vineyard, only Copper (Cu) treatment is allowed, like antifungal. The aim was to investigate the chemical and biochemical properties of the soil with geese and the impact of Cu on the soil and animal tissues. The main results showed that the presence of animals improves the efficiency of the microbial biomass mainly in the upper soil horizons. Moreover, the grazing activity of geese removes Cu from the soil with the grass intake and showed a moderate accumulation in the liver. However, no significant difference was present in the edible tissues (breast and drumstick) of the vineyard geese in respect to the control ones.

**Abstract:**

Agroforestry systems aim at increasing the productivity and the environmental sustainability of both crop and animal productions. The integration of small animals such as geese in the vineyard could represent an opportunity to improve farm income and reduce land use for grazing. The main objective of this work was to study the impact of geese rearing in an organic vineyard on the chemical and biochemical properties of the soil and the effect of Copper (Cu) supplied with the fungicide treatments. Furthermore, the amount of Cu in the animal tissues was also investigated. Three experimental areas within the vineyard were selected: High Geese Density (HGD-240 geese ha^−1^), Low Geese Density (LGD-120 geese ha^−1^) and Without Geese used as control soil (WG). The results indicated that both HGD and LGD did not affect the main chemical properties of the vineyard soils. LGD increased the amount and the efficiency of the microbial biomass in the upper soil horizons. Moreover, geese through the grazing activity reduced the Cu content in the vineyard soils, accumulating this element in their liver. However, the content of Cu in the breast and drumstick of vineyard geese did not show any significant difference in respect the meat of the control ones.

## 1. Introduction

Pasture-based animal productions are considered with great interest due to their positive effects on meat quality and animal welfare and health [1,2,3], but the environmental impact of these systems is still debated because of the high land use for grazing [4]. In a previously study, Cartoni Mancinelli et al. [3] showed that the geese grazing activity at a vineyard had a positive effect on the meat quality improving tocopherols, retinol and Long Chain Pufa of n-3 series (LCPn-3) content.

Moreover, the combination of perennial crops (such as orchards, vineyards or olive groves) and animals in the same area eliminates additional land needed for grazing. This integration aims at increasing the productivity and the environmental sustainability [5] of both crop and animal production. The integrated crop/livestock agriculture permits to diversify the agroecosystem and increase ecosystem services such as crop production, farm economy, weed, pest control and soil fertility [6,7,8,9,10,11]. Indeed, animals recycle nutrients contained in forage and feedstuff and make them available in their excreta, thus becoming part of the on-farm nutrient cycle. The amount of nutrients (i.e., N, P, K) supplied to soil through animal manure largely differ among species, depending on the foraging preferences of the animal as well as the supplemental feedstuff provided [12]. When properly managed, animal droppings can provide organic matter, macronutrients and trace elements fundamental for the crops, the activity of the soil microbial community [10], and decrease the need for external fertilizers. Several studies assessed an improvement of the soil quality under integrated crop/livestock management [13,14,15].

In Europe, vineyards occupy 4.563 million hectares, with Spain, France and Italy covering most of this large surface [16]. Vineyards also cover large areas outside Europe mainly in Asia, USA and the Southern Hemisphere [17]. Considering the worldwide diffusion of grape cultivation, there is an increasing need for sustainable practices supporting soil fertility of this agroecosystem. In this view, integrating livestock into vineyards, and in particular small animals such as geese, can represent an opportunity to integrate sustainability and farm income [18] and reduce land use for grazing [19].

Geese rearing into the vineyard has countless benefits: (i) animals eat grass and young weeds as quickly as they appear; (ii) they eat grass and weeds next to plants that cannot be removed by hoeing or tillage. However, animal density is crucial and geese over-grazing can potentially damage and could produce soil compaction, and vice-versa if it is too low may not be effective in weeding [20]. Naturally, when the animals are raised in orchards, only some biocide pesticides should be used for avoiding residue in the animal products. Copper (Cu) is one of the most important fungicides used in organic farms. Concerning human health, Cu is an essential mineral but at the same time can be toxic depending of the amounts ingested. Warning and critical legislative limits valid in the European Union (EU) set Cu concentrations in soils to 50 and 140 mg kg^−1^ (Council Directive 86/278/EC,1986), due to its impact on the environment; however, at this stage, concrete and robust solutions do not exist for replacing current Cu-based fungicides.

With this background, the main objective of the work was to study the impact of geese rearing in a vineyard on the chemical and biochemical properties of the soil. Furthermore, a deepening on the Cu cycle in the environment (soil and grass) and in the bodies of geese (liver and meat) was investigated.

## 2. Materials and Methods

### 2.1. Site Description

The study was conducted during the year 2014 in the vineyard of a farm located in the municipality of Cannara (Perugia, central Italy, 42°59′19.78″ N–12°33′00.41″ E) at about 250 m a.s.l. The vineyard was organic; accordingly, the only allowed treatment to fight mildew was by spraying Cu-based fungicide [copper oxychloride, Cu_2_(OH)_3_Cl]. The climate of the area is continental, the mean annual air temperature is 13.8 °C, with January as the coldest (4.7 °C) and July as the warmest (22.9 °C) month, and the mean annual precipitation is 864 mm.

The soils of the studied site developed from fluvial and lacustrine sediments had a clay-loam texture, and they were classified as mixed, mesic Typic Haplustepts (Soil Survey Staff, 2014) [21]. 

### 2.2. Geese Grazing

Within the vineyard (cultivar *Grechetto* and *Trebbiano Spoletino*), whose alleys were left to spontaneous colonization of herbaceous species, three sites were selected: one with High Geese Density (HGD) (240 geese ha^−1^), another with Low Geese Density (LGD) (120 geese ha^−1^) and a third Without Geese and used as control soil (WG). The three areas were about 0.5 ha for HGD, 1 ha for LGD and 1 ha for WG. Our trial was conducted according to EU Regulation 834/07, EU Regulation 889/2008 and Italian directives (Gazzetta Ufficiale 1992) on animal welfare for experimental and other scientific purposes and not required any ethical approval. 

In two consecutive years (2013–2014), geese ducklings of *Romagnola* breed were purchased in mid-February. One-day-old geese were reared under brooder lamps for the next 3 weeks. The environmental temperature ranged from 20 to 25 °C and the relative humidity from 65% to 75%. At 21 days of age, the geese were divided into three groups: 120 animals reared in conventional conditions without pasture (Control group) and two experimental groups of 120 and 240 geese which had access to the vineyard (LGD and HGD, respectively). They were housed indoor up to about the middle of April and, then, they were moved to the experimental areas within the vineyard. In this period, the vine branches were sufficiently developed (more than 30 cm in length), so that the goose chicks would not cause damage to the vineyard. In the vineyard, rows were separated into sectors to allow more efficient grazing by geese. 

The geese were fed additional feedstuff (40% corn, 30% wheat and/or barley and 30% faba bean) supplied each day at evening, while water was provided ad libitum. During the night, the geese were placed into huts made of welded mesh for protection from predators. The rearing period of the geese into the vineyard was about 120 days per year. To estimate the forage intake, the modified method of Lantinga et al. [22] was applied. Five metallic fences (0.50 × 0.50 m) per pen were positioned at about 20 m from each hut, in each area. For each replication, herbage samples were collected at the beginning (outside the exclusion pens) and at the end (both inside and outside the exclusion pens) of the rearing cycle. Forage intake (FI) was estimated using the following equation [23]:

FI = (GMs − GMe) + {[1 − (GMe/GMs)]/− ln[GMe/GMs]} × (GMu − GMs),(1)

where: GMs = herbage mass present at the entrance of the geese in each pen; GMe = forage that remained at the end of the trial; and GMu = undisturbed forage mass from a nearby ungrazed area.

The amount of dropping per goose was estimated according to Kear [24] and the amount of Nitrogen (N), Phosphorus (P), Potassium (K) and Carbon (C) in the geese dropping was analyzed according to Official Methods of Analysis (AOAC) [25] methods.

### 2.3. Soil Sampling

In each study area, during fall of the year 2014, the pedological variability was evaluated by a preliminary survey and by opening several auger holes and mini-pits. Once the limited soil heterogeneity was assessed, in each area (HGD, LGD, WG) two pedological profiles were dug, morphologically described according to Schoeneberger et al. [26] (see Table S1, supplementary materials), and the upper soil horizons (Ap1 and Ap2) sampled.

The soil samples collected for each horizon at each profile of the experimental sites (HGD, LGD, WG) were used as replicates (*n* = 2). An aliquot of each soil sample was sieved through a 4-mm mesh at field moist conditions and stored for a period not exceeding two weeks at 2 °C for the biological analyses: microbial biomass C content, basal respiration and microbial community structure. The remaining sample aliquots were air-dried and sieved at 2 mm and used for chemical analyses.

### 2.4. Chemical Soil Analysis

The soil pH was determined potentiometrically in water (solid:liquid ratio of 1:2.5) after one night of equilibration using a Thermo Scientific™ Orion™ 2-Star Benchtop pH-meter. The total organic C (TOC) was estimated by K-dichromate digestion method, heating the suspension at 180 °C for 30 min [27]. The water extractable organic C (WEOC) was extracted by mixing 1 g of soil with 10 mL of water. The mixture was shaken overnight with an orbital shaker (140 rpm), centrifuged at 1400 g for 10 min and filtrated through a 0.45 μm membrane filter [28], and its organic C content was analyzed by K-dichromate digestion method, as reported above. The total N (TN) was measured by a dry combustion analyzer (EA-1110, Carlo Erba Instruments, Milan, Italy). The inorganic N forms (NH_4_^+^-N and NO_3_^−^-N) were determined, after processing the samples with 2 M KCl solution (solid:liquid ratio 1:10), by a FOSS Fiastar™ 5000 system (Hillerod, Denmark). The difference between the total N and inorganic N content was considered as organic N.

### 2.5. Soil Microbial Biomass C and Basal Respiration

The fumigation-extraction method was used for the determination of the amount of the soil microbial biomass-C (C_mic_) [29], after 62 days of incubation at 25 °C and at 50% of soil total water holding capacity. During the incubation period, basal respiration was estimated by alkali (1 M NaOH solution) absorption of the evolved CO_2_ by back-titration of the residual OH^−^ with a standardized HCl solution and expressed as the cumulative amount of CO_2_-C developed during the experiment.

### 2.6. Soil Microbial Community Structure

The characterization of the microbial community structure in the soil samples was assessed by analyzing the ester-linked phospholipid fatty acids (PLFAs), which are retained as an indicator of living biomass. The extraction, fractionation and quantification of lipids were performed from 2 g of fresh soil samples, following the procedure described by Bardgett et al. [30]. Finally, fatty acid methyl esters were detected by an Agilent 7890-A gas-chromatograph, equipped with a 5975C MSD detector and Agilent HP-Innowax column (50 m, 0.20 mm I.D., 0.40 mm D.F.). Separated fatty-acid methyl-esters were identified by chromatographic retention time and mass spectral comparison using the BAME mix qualitative standard (Supelco Analytical, Bellefonte, PA, USA). The concentration of each PLFA was calculated by comparing the peak area of each identified fatty acid with that of methyl nonadecanoate (C19:0) added to the samples as an internal standard. The recognized PLFAs were used as markers to quantify the relative abundance of specific cell types [31,32]. Gram-positive bacteria (Gram+) were identified by summing i15:0, a15:0, i16:0, i17:0 and a17:0 fatty acids, while the Gram-negative bacteria (Gram-) were accounted by summing the fatty acids 16:1, cy17:0, 17:1ω9c and 18:1ω7 [30,32,33,34]. The total bacterial biomass was calculated by the sum of the PLFAs assigned to Gram+ and Gram- bacteria. The fatty acid 18:2ω6 was used as a marker for saprophytic fungi [33], while the fatty acid 16:1ω5 was attributed to arbuscular mycorrhizal fungi (AMF) [35]. This latter acid is not strictly specific to AMF, although it was often used as an indicator for their abundance in soil [35,36,37]. The 10Me17:0 and 10Me18:0 fatty acids were assigned to Actinomycetes [35,38], whereas the 20:2 fatty acid was used as biomarker for protozoa [32]. 

### 2.7. Cu Determination in Soil, Grass and Animal

The soil and grass from each area (HGD, LGD, WG) were specifically sampled in late spring, after the end of Cu-based fungicide treatments. The samples were dried in an oven at 80 °C and the grass samples subdivided into aerial parts and roots. 

Regarding the animals, at 180 days old, 10 geese from each group (HDG, LDG and control animals from geese with no access to pasture) were slaughtered. Samples were taken from the livers, breasts and drumsticks and freeze-dried. 

An aliquot (0.4 g) of each sample was microwave digested (ETHOS One high-performance microwave digestion system; Milestone Inc., Sorisole, Bergamo, Italy) with 8 mL of ultrapure concentrated nitric acid (65% w/w, Carlo Erba, Milan, Italy) and 2 mL of hydrogen peroxide (30% w/w, Carlo Erba, Milan, Italy), and heat of 200 °C was applied for 30 min. Cu concentrations were determined by flame atomic absorption spectrophotometry using a Shimadzu AA-6800 apparatus (Shimadzu Corp., Tokyo, Japan).

### 2.8. Statistical Analysis

Two-way ANOVA was used to compare chemical and biological soil properties as a function of geese management, and soil horizons. The geese traits (feed, grass, Cu content of liver, breast and drumstick) were analyzed with one-way ANOVA comprising the fixed effect of geese density (HGD and LGD). Homogeneity of variances was verified by graphical analysis of residuals. When the normality and homoscedasticity was not satisfied, the logarithmic transformation was selected by the maximum likelihood procedure devised by Box and Cox [39], as implemented in the box cox function of the package Modern Applied Statistics with S (MASS) [40] in the R statistical environment [41]. All significant effects were assessed by Tukey post-hoc test at *p* = 0.05.

## 3. Results and Discussion

### 3.1. Soil Properties

The pH values of the three sites were generally sub-alkaline, ranging from 7.64 to 7.97 (Table 1), because of the soils developed from fine textured and carbonate rich layers that exert a buffering capacity against the protons and organic ligands released by roots [42]. However, the pH values of the upper horizons of HGD and LGD were higher than WG presumably as a result of goose dropping release on soil (mean pH of geese dropping = 7.88 ± 0.01). 

Conversely to our hypothesis, and although many authors reported that repeated applications of organic waste such as animal manure, municipal waste and sewage sludge increase the soil organic matter content [43,44], the presence of geese did not enrich TOC and WEOC content in the HGD and LGD with respect to the WG (Table 1). This lack of TOC and WEOC increase was attributed to the fact that the potential organic carbon input to the soil due to the geese droppings (Table 2) was counterbalanced by

(i) the reduction of the input deriving from the grass cover due to the geese grazing (about 259 and 129 kg C/ha 100 d, respectively in HGD and LGD);
(ii) a possible degradation of the geese droppings, which remains on the soil surface with a consequent loss of C in form of CO_2_ emission toward the atmosphere.

Contrary to expectation, TOC content does not increase in LGD and HGD soil with respect to WG. This fact could partially due to a priming effect occurring as a consequence of the addition of easily degradable organic substances to soil [45,46]. 

Indeed, the geese droppings had a high WEOC content (mean WEOC content of goose feces =18.4 ± 0.8 g kg^−1^d.w.) that represent an energy source for the soil microbiota, triggering the shift from a dormant to an active state of growth of the soil microbial community [47,48] and the mineralization of stabilized organic matter [49]. However, our study design could not assess soil chemical and microbiological modifications underlying the above-mentioned priming effect that generally take place during the first few weeks after the application of organic substrata [45].

Total N content, which was mostly comprised by organic N, was similar along HGD, LGD and WG profiles (Table 3). This fact was attributed to a balanced amount of N added to the soil with the droppings and removed from the soil with the geese grazing (Table 2). In all the samples, NO_3_^−^-N represented the smallest portion of the total soil N (Table 3). The significantly greater amount of NO_3_^—^N content in the Ap1 horizon of HGD than that of LGD and WG (Table 3), suggested that the high animal density was able to increase the main form of nitrogen available to plants in the upper soil horizon.

It is well known that both urine and feces of herbivores provide highly decomposable resources that are rich in labile nutrients able to stimulate both plant N acquisition and growth in a wide range of natural and semi-natural ecosystems [50,51] and soil microbial biomass [52,53,54]. Our results were in accordance with the latter authors, indeed we found that the microbial biomass C content (Table 4) was higher in the HGD and LGD with respect to WG. Conversely, the ΣCO_2_-C was significantly lower in the Ap horizons of HGD and LGD than in that of WG. These results are not in accordance with Carvalho et al. [55], which reported that microbial biomass and basal respiration were stimulated with increasing grazing intensity due to a higher pasture root mass at the end of the pasture phase in a crop-livestock experiment in southern Brazil. 

In our experiment the large extent of microbial biomass C, together with the low CO_2_-C evolved during the basal respiration experiment, suggested a better adaptation of the microbial community hosted in the HGD and LGD than that of WG. Further, the greater C_mic_/TOC ratio in the LGD than that in WG indicated a higher substrate-use efficiency of the microbial community [56] in the vineyard soil with the low animal density.

**Figure 1:**
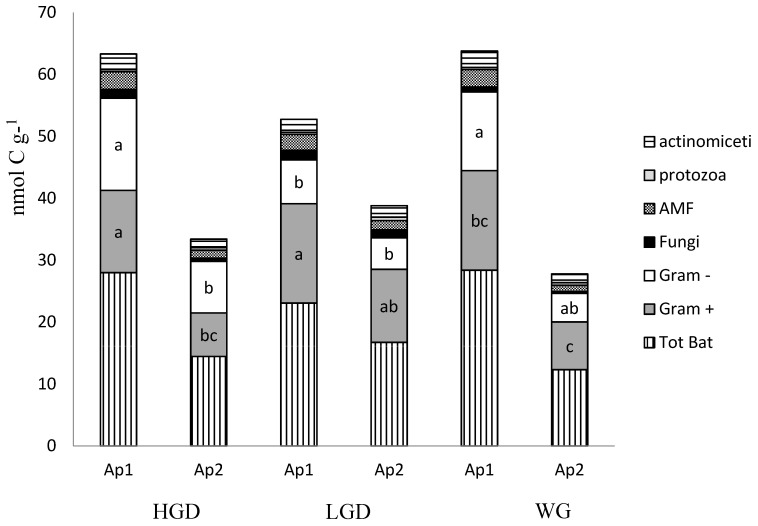
Main microbial community estimated by Ester-linked phospholipid fatty acids (PLFA) in soils with a high (HGD) and low (LGD) geese density, and control vineyard (WG). Different soil horizons are compared (Ap1, Ap2) of the vineyard. For each histogram, different letters significantly differ for *p* < 0.05.

In all the vineyard soils, bacteria were the most represented microbial group identified by PLFAs ranging from 76% (HGD Ap1) to 36% (WG Ap1) of the entire microbial community (Figure 1). 

Within the microbial community, bacteria were the most abundant group, followed by *Actinomycetes*, AMF, saprophytic fungi and protozoa. Our results indicated that two years of geese grazing affected the structure of the bacterial community, mainly in the upper horizon. In particular, the Gram+ bacteria, inhabiting the Ap1 horizon, were more abundant in both HGD and LGD than in WG, whereas the Gram- bacteria were less copious in the Ap1 of LGD than in the HGD and WG. Consequently, LGD showed the highest Gram+/Gram- ratios (Table 4). This shift toward Gram+ dominated bacterial community that occurred in the upper horizon of both HGD and LGD could be due to the exogenous organic matter added to soil by the geese droppings, which host a specific microbial community. Lu et al. [57], performing analyses of several goose fecal clones from Ontario and Ohio, found that goose fecal communities are dominated by *Clostridia* (represented by 33.7% of clones) and *Bacilli* (38.1% of clones), and by the phylum *Bacteroidetes* (10.1% of clones). The main species of *Clostridia* are often, but not always, Gram+ [58], and *Bacilli* are almost exclusively Gram+; conversely, the *Bacteroidetes* phylum is composed of three large classes of Gram- [59]. The research conduct by Lu et al. [57] showed that about 70% of the goose fecal microbial community consisted of Gram+ bacteria.

### 3.2. Copper Cycle in the System Soil-Grass-Geese

In this agroforestry system the Cu cycle was triggered by the vineyard managements. Indeed, the vines are treated several times per year with copper oxychloride as fungicide. The Cu added with treatments is partly absorbed by the vine leaves, while another fraction drips and/or is leached by rainwater from the vine canopies to grass and soil. Sometimes Cu accumulation in vineyard soils reaches phytotoxic thresholds [60]. In our case, the greater amount of Cu both in the vineyard grazing with geese than control vineyard, was in the upper soil horizons due to the limited mobility of this metal in soils, as generally reported by different authors [61,62] (Table 5). Indeed, Cu in soils is strongly fixed mainly by organic matter [63,64]. 

With regard to the grass, greater Cu accumulation occurred in the roots than in the leaves suggesting a limited translocation of this metal inside the plant tissue with no difference between control (WG) and geese vineyard (LDG and HGD). However, due to the Cu-based antifungal treatments the grass had a higher concentration of Cu (about 11.1 mg kg^−1^, Table 5) with respect to the feed (8.0 mg kg^−1^).

Our data suggested that the presence of the geese reduced the Cu content of the soil (Table 5) through grazing, as the Cu ingested with the grass by the animals is removed from the system and only part returned to soil in the less available form (organic Cu in feces).

There is a significant difference between the estimated intake of Cu in the two groups (Table 6). In particular, in both the vineyard with grazing geese (HGD and LGD) there was about 3.3-fold higher intake of Cu than control. Consequently, this higher Cu intake of the vineyard groups significantly increased the concentration of Cu in their liver (152 and 144 vs. 95 mg kg^−1^). The increase of Cu level in liver, although relevant, is not dangerous for animals because in other trials several authors found an even higher Cu amount without any sign of toxic effect. Magali et al. [65] found a high variability of Cu concentration in the liver (between 168 and 540.4 mg/kg of DW) in different duck genotypes not overfeed.

Chiou et al. [66] investigated about the supplementation of 200, 400, 600 and 800 mg kg^−1^ of Cu in diet of laying hens. The liver enzymes activity (AST, LDH, and CK) significantly increased only with 600 mg of dietary Cu.

Compared to these studies, the Cu intake of vineyard geese was very low, and both the vineyard geese excreted more than 90% of Cu intake with faeces; Skřivan, et al. [67] demonstrated that the supplementation of poultry diets with increased concentrations of Cu linearly enlarges the concentration of Cu in excreta from 25.3 to 396.8 mg kg^−1^d.m.

However, in the breast and drumstick meat samples there was no significant difference between the Cu content of the control group and the vineyard group.

Our results are in line with Bortey-Sam et al. [68] who have observed that Cu accumulation was more pronounced in chicken liver and kidney than in muscles.

Falandysz [69] compared the liver Cu content of different species and found that geese show a higher value of Cu compared to turkey, chicken, hen, rabbit and sheep. 

## 4. Conclusions

The results of this study indicated that the geese seem to be able to perform good weeding and fertilizing of the vineyard without damage to the soil. Moreover, both goose densities (HGD and LGD) did not affect the main chemical properties of the vineyard soils, although the lower animal load seemed to increase the amount and the efficiency of the microbial biomass in the superficial horizons, which could be considered as an improvement of the soil quality. 

The presence of geese in the vineyard contributed to the reduction of the Cu content in the soils through the grass grazing, leading to an accumulation of this element in the liver. However, this level is much lower than the quantity retained safe (0.15 mg/kg bw/d Commission of the European Communities), and no significant difference was detected for the Cu content in breast and drumstick meat between geese reared under the vineyard and control group.

Once further developed, geese farming in vineyards could be an example of economic-ecological reconciliation, combining increased productivity per hectare of land with environmental sustainability. 

Moreover, the consumer attention toward more sustainable products with lower environmental impact opens the way to develop alternative production systems such as geese in the vineyard.

## Figures and Tables

**Table 1:** Values of pH, and contents of total organic C, and water extractable organic C of the vineyard soils with a high and low geese density, and of the soil of the control vineyard. Numbers in parentheses are the standard errors (*n* = 2).

SITE ^1^	pH	TOC ^2^	WEOC ^3^
Horizons		g kg^−1^	
HGD			
Ap1	7.85 (0.00) ^c^	14 (2) ^a^	0.4 (0.1) ^a^
Ap2	7.97 (0.01) ^a^	9.9 (0.4) ^ab^	0.53 (0.02) ^a^
LGD			
Ap1	7.88 (0.00) ^bc^	9.9 (0.3) ^ab^	0.45 (0.03) ^a^
Ap2	7.92 (0.02) ^ab^	6.6 (0.7) ^b^	0.36 (0.01) ^b^
WG			
Ap1	7.64 (0.01) ^d^	12.9 (0.1) ^a^	0.38 (0.08) ^a^
Ap2	7.83 (0.01) ^c^	9.2 (0.9) ^ab^	0.37 (0.08) ^a^

^1^ HGD: High Goose Density (240 geese ha^−1^), LGD: Low Goose Density (120 geese ha^−1^), WG: control soil without geese; ^2^ TOC: total organic carbon; ^3^ WEOC: water extractable organic carbon. Ap1, Ap2 different soil horizons. Within each column, different letters indicate significant differences between means (*p* ≤ 0.05).

**Table 2:** Calculated amount (kg d.m./ha) of N, P, and C added to soil with the geese droppings (Input) and take out by the herbage grazing (Intake) during the rearing period (120 days).

Compounds ^1^		K	N	P	C
	SITE ^2^	kg d.m./ha			
Input	HGD	18.7	43.05	18.72	1310
	LGD	9.3	21.52	9.36	655
Intake	HGD	-	45.28	9.81	259
	LGD	-	22.64	4.90	129

^1^ K: potassium, N: nitrogen, P: phosphorus, C: carbon; ^2^ HGD: High Goose Density (240 geese ha^−1^); LGD: Low Goose Density (120 geese ha^−1^).

**Table 3:** Contents of total N, ammonium and nitrate of the vineyard soils with a high and low geese density, and of the soil of the control vineyard. Numbers in parentheses are the standard errors (*n* = 2).

SITE ^1^	Compounds ^2^			
	Total N	NH_4_^+^-N	NO_3_^−^-N	Organic N
Horizons	g kg^−1^	mg kg^−1^	mg kg^−1^	g kg^−1^
HGD				
Ap1	0.9 (0.1) ^a^	27 (1) ^b^	6.5 (0.1) ^a^	0.9 (0.1) ^a^
Ap2	0.8 (0.1) ^a^	23.8 (0.5) ^b^	0.74 (0.06) ^c^	0.8 (0.1) ^a^
LGD				
Ap1	1.08 (0.03) ^a^	23.6 (0.7) ^b^	0.81 (0.02) ^c^	1.06 (0.03) ^a^
Ap2	0.98 (0.01) ^a^	23 (2) ^b^	1.75 (0.09) ^b^	0.96 (0.02) ^a^
WG				
Ap1	1.15 (0.06) ^a^	27 (1) ^b^	2.6 (0.3) ^b^	1.12 (0.06) ^a^
Ap2	1.16 (0.05) ^a^	41 (2) ^a^	7 (2) ^a^	1.11 (0.05) ^a^

^1^ HGD: High Goose Density (240 geese ha^−1^); LGD: Low Goose Density (120 geese ha^−1^); WG: control soil without geese; ^2^ total N: total nitrogen, NH_4+_-N: ammonium, NO_3_—N: nitrate, organic N: organic nitrogen. Ap1, Ap2 different soil horizons. Within each column, different letters indicate significant differences between mean (*p* ≤ 0.05).

**Table 4:** Content of microbial biomass C, amount of CO_2_ evolved during basal respiration experiments, and Cmic/TOC ratio of soils with high and low geese density, and of the soil of the control vineyard. Numbers in parentheses are the standard errors (*n* = 2).

SITE ^2^	Cmic	ΣCO_2_-C	Cmic/TOC Ratio
Horizons	mg kg^−1^	mg kg^−1^	
HGD			
Ap1	1705 (213) ^ab^	420 (11) ^c^	0.12 (0.00) ^ab^
Ap2	1255 (58) ^ab^	584 (15) ^b^	0.13 (0.00) ^ab^
LGD			
Ap1	2229 (453) ^a^	644 (16) ^b^	0.22 (0.05) ^a^
Ap2	1827 (170) ^a^	315 (2) ^d^	0.27 (0.06) ^a^
WG			
Ap1	1041 (113) ^bc^	875 (32) ^a^	0.08 (0.01) ^b^
Ap2	796 (19) ^c^	326 (2) ^d^	0.09 (0.01) ^b^

Cmic: microbial biomass of carbon, ΣCO_2_-C: amount of CO_2_ evolved during basal respiration experiments, and Cmic/TOC ratio: microbial biomass of carbon/total organic carbon; ^2^ HGD: High Goose Density (240 geese ha^−1^); LGD: Low Goose Density (120 geese ha^−1^); WG: control soil without geese. Ap1, Ap2 different soil horizons. Within each column, different letters indicate significant differences between mean (*p* < 0.05).

**Table 5:** Copper concentration in the feed, soil (horizons Ap1 and Ap2), and different plant portions (mg/kg) of the vineyard soils with a high and low geese density, and of the soil of the control vineyard. Numbers in parentheses are the standard errors (*n* = 2).

Site ^1^	LGD	HWG	WG
Feed	8.0 (1)	8.0 (1)	8.0 (1)
Soil Ap1	45 (2)	39 (3)	58 (3)
Soil Ap2	40 (3)	36 (3)	52 (5)
Roots	25 (1)	27 (1)	22 (2)
Grass	11 (0.9)	11 (0.8)	11 (0.5)

^1^ HGD: High Goose Density (240 geese ha^−1^); LGD: Low Goose Density (120 geese ha^−1^); WG: control soil without geese.

**Table 6:** Estimated copper intake and copper content in tissues and feces of geese of reared at high and low geese density, and control group. Numbers in parentheses are the standard errors (*n* = 2).

Traits	Unit of Measure	LGD	HGD	Control
Estimated Cu intake	mg/d	4 (1)	3 (1)	1 (0.9)
Liver	mg kg^−1^	152 (5) ^b^	144 (5) ^b^	95 (3) ^a^
Breast meat	mg kg^−1^	3 (1)	3 (1)	3 (1)
Drumstick meat	mg kg^−1^	1 (1)	1 (0.8)	0.9 (0.5)
Feces	mg kg^−1^	59 (4) ^b^	55 (3) ^b^	23 (3) ^a^

HGD: High Goose Density (240 geese ha^−1^); LGD: Low Goose Density (120 geese ha^−1^); Control: geese reared out of the vineyard.

## Supplementary Materials

The following are available online at https://www.mdpi.com/2076-2615/9/4/179/s1, Table S1: Morphological description by Schoeneberger et al. (2012) of the profiles of the vineyard soils with a High (HGD) and Low (LGD) Geese Density, and of the soil of the control vineyard Without Geese (WG), Cannara (PG, central Italy).

## References

[1] Fraser D. (2006). Animal welfare assurance programs in food production: A framework for assessing the options. Anim. Welf..

[2] Dal Bosco A., Mugnai C., Mattioli S., Rosati A., Ruggeri S., Ranucci D., Castellini C. (2016). Transfer of bioactive compounds from pasture to meat in organic free-range chickens. Poult. Sci..

[3] Cartoni Mancinelli A., Mattioli S., Dal Bosco A., Piottoli L., Ranucci D., Branciari R., Cotozzolo E., Castellini C. (2019). Rearing Romagnola geese in vineyard: Pasture and antioxidant intake, performance, carcass and meat quality. Ital. J. Anim.Sci..

[4] Phelps L.N., Kaplan J.O. (2017). Land use for animal production in global change studies: Defining and characterizing a framework. Glob. Chang. Biol..

[5] Patrizi N., Niccolucci V., Castellini C., Pulselli F.M., Bastianoni S. (2018). Sustainability evaluation of agro-livestock integration: Implications and results of Emergy evaluation. Sci. Total Environ..

[6] Clark A.E. (2004). Benefits of re-integrating livestock and forages in crop production systems. J. Crop Improv..

[7] Hanson J.D., Franzluebbers A. (2008). Principles of integrated agricultural systems. Renew. Agric. Food Syst..

[8] Hendrickson J.R., Hanson J.D., Tanaka D.L., Sassenrath G. (2008). Principles of integrated agricultural systems: Introduction to processes and definition. Renewable Agric. Food Syst..

[9] Hendrickson J.R., Liebig M.A., Sassenrath G. (2008). Environment and integrated agricultural systems. Agric. Food Syst..

[10] Russelle M.P., Entz M.H., Franzluebbers A.J. (2007). Reconsidering integrated crop-livestock systems in North America. Agron. J..

[11] Tanaka D.L., Anderson R.L., Rao S.C. (2005). Crop sequencing to improve use of precipitation and synergize crop growth. Agron. J..

[12] Watson C.A., Öborn I., Eriksen J., Edwards A.C. (2005). Perspectives on nutrient management in mixed farming systems. Soil Use Manag..

[13] Acosta-Martínez V., Zobeck T.M., Allen V. (2004). Soil Microbial, Chemical and Physical Properties in Continuous Cotton and Integrated Crop–Livestock Systems. Soil Sci. Soc. Am. J..

[14] Maughan M.W., Flores J.P.C., Anghinoni I., Bollero G., Fernandez F.G., Tracy B.G. (2009). Soil quality and corn yield under crop-livestock integration in Illinois. Agron. J..

[15] Lowy P. (2009). Integrating poultry and sheep on vegetable cropping land for increased economic return and enhanced fertility. Sustainable Agriculture Research and Education Project Database.

[16] FAO (2011). FAOSTAT. Commodities by Country. http://faostat/fao.org/.

[17] OIV (International Organization of Vine and Wine) Statistical Report on World Viticulture 2012. http://www.oiv.int.

[18] Hilimire K. (2011). Integrated Crop/Livestock Agriculture in the United States: A Review. J. Sustain. Agric..

[19] Paolotti L., Boggia A., Castellini C., Rocchi L., Rosati A. (2016). Combining livestock and tree crops to improve sustainability in agriculture: A case study using the Life Cycle Assessment (LCA) approach. J. Clean. Prod..

[20] Abdalla M., Hastingsa A., Chadwick D.R., Jones D.L., Evans C.D., Jones M.B., Rees R.M., Smith P. (2018). Critical review of the impacts of grazing intensity on soil organic carbon storage and other soil quality indicators in extensively managed grasslands. Agric. Ecosyst. Environ..

[21] Soil Survey Staff (2014). Keys to Soil Taxonomy.

[22] Lantinga E.A., Neuteboom J.H., Meijs J.A.C., Penning P.D. (2004). Sward methods. Herbage Intake Hand Book.

[23] Dal Bosco A., Mugnai C., Rosati A., Paoletti A., Caporali S., Castellini C. (2014). Effect of range enrichment on performance, behavior, and forage intake of free-range chickens. J. Appl. Poult. Res..

[24] Kear J. (1963). The agricultural importance of wild goose droppings. Wildfowl.

[25] AOAC (1995). Official Methods of Analysis of the AOAC International.

[26] Schoeneberger P.J., Wysocki D.A., Benham E.C. (2012). Soil Survey Staff. Field Book for Describing and Sampling Soils.

[27] Nelson D.W., Sommers L.E., Sparks D.L. (1996). Total carbon, organic carbon, and organic matter. Methods of Soil Analysis, Part 3. Chemical Methods.

[28] Agnelli A., Bol R., Trumbore S.E., Dixon L., Cocco S., Corti G. (2014). Carbon and nitrogen in soil and vine roots in harrowed and grass-covered vineyards. Agric. Ecosyst. Environ..

[29] Vance E.D., Brookes P.C., Jenkinson D.S. (1987). An extraction method for measuring microbial biomass C. Soil Biol. Biochem..

[30] Bardgett R.D., Hobbs P.J., Frostegård Å. (1996). Changes in soil fungal:bacterial biomass following reductions in the intensity of management of an upland grassland. Biol. Fertil. Soils.

[31] Frostegård Å., Bååth E., Tunlid A. (1993). Shifts in the structure of soil microbial communities in limed forests as revealed by phospholipid fatty acid analysis. Soil Biol. Biochem..

[32] Fritze H., Pietikainen J., Pennanen T. (2000). Distribution of microbial biomass and phospholipid fatty acids in Podzol profiles under coniferous forest. Eur. J. Soil Sci..

[33] Fierer N., Schimel J.P., Holden P.A. (2003). Variation in microbial community composition through two soil depth profiles. Soil Biol. Biochem..

[34] Federle T.W., Megusar F., Gantar M. (1986). Microbial distribution in soil new techniques. Perspectives in Microbial Ecology.

[35] Massaccesi L., Benucci G.M.N., Gigliotti G., Cocco S., Corti G., Agnelli A. (2015). Rhizosphere effect of three plant species of environment under periglacial conditions (Majella Massif, central Italy). Soil Biol. Biochem..

[36] De Deyn G., Quirk H., Bardgett R. (2011). Plant species richness, identity and productivity differentially influence key groups of microbes in grassland soils of contrasting fertility. Biol. Lett..

[37] Olsson P.A. (1999). Signature fatty acids provide tools for determination of the distribution and interactions of mycorrhizal fungi in soil. FEMS Microbiol. Ecol..

[38] Chung H., Zak D.R., Reich P.B., Ellsworth D.S. (2007). Plant species richness, elevated CO_2_, and atmospheric nitrogen deposition alter soil microbial community composition and function. Glob. Chang. Biol..

[39] Kroppenstedt R.M., Goodfellow M., Minnikin D.E. (1985). Fatty acid and menaquinone analysis of actinomycetes and related organisms. Chemical Methods in Bacterial Systematics, Society for Applied Bacteriology (Technical Series No. 20).

[40] Box G.E.P., Cox D.R. (1964). Analysis of transformations. J. R. Stat. Soc. Ser. B Stat. Methodol..

[41] Venables W.N., Ripley B.D. (2002). Modern Applied Statistics with S.

[42] R Core Team (2014). A Language and Environment for Statistical Computing. R Foundation for Statistical Computing.

[43] Agnelli A., Massaccesi L., De Feudis M., Cocco S., Courchesne F., Corti G. (2016). Holm oak (*Quercus ilex* L.) rhizosphere affects limestone-derived soil under a multi-centennial forest. Plant Soil.

[44] Khaleel R., Reddy K.R., Overcash M.R. (1980). Transport of potential pollutants in runoff water from land areas receiving animal wastes: A review. Water Res..

[45] Scotti R., Bonanomi G., Scelza R., Zoina A., Rao M.A. (2015). Organic amendments as sustainable tool to recovery fertility in intensive agricultural systems. J. Soil Sci. Plant Nutr..

[46] Blagodatskaya E., Kuzyakov Y. (2008). Mechanisms of real and apparent priming effects and their dependence on soil microbial biomass and community structure: Critical review. Biol. Fertil. Soils.

[47] Guenet B., Neill C., Bardoux G., Abbadie L. (2010). Is there a linear relationship between priming effect intensity and the amount of organic matter input?. Appl. Soil Ecol..

[48] De Nobili M., Contin M., Mondini C., Brookes P.C. (2001). Soil microbial biomass is triggered into activity by trace amounts of substrate. Soil Biol. Biochem..

[49] Drake J.E., Darby B.A., Giasson M.A., Kramer M.A., Phillips R.P., Finzi A.C. (2012). Stoichiometry constrains microbial response to root exudation—Insights from model and a field experiment in a temperate forest. Biogeochem. Discuss..

[50] Marinari S., Masciandaro G., Ceccanti B., Grego S. (2000). Influence of organic and mineral fertilisers on soil biological and physical properties. Bioresour. Technol..

[51] McNaughton S.J., Banyikwa F.F., McNaughton M.M. (1997). Promotion of the cycling of diet-enhancing nutrients by African grazers. Science.

[52] Frank D.A., Groffman P.M. (1998). Ungulate vs. Landscape control of soil c and n processes in grasslands of Yellowstone national park. Ecology.

[53] Frank D.A., Evans R.D. (1997). Effects of native grazers on grassland N cycling in Yellowstone National Park. Ecology.

[54] Bardgett R.D., Wardle D.A., Yeates G.W. (1998). Linking above-ground and below-ground interactions: How plant responses to foliar herbivory influence soil organisms. Soil Biol. Biochem..

[55] Bardgett R.D., Wardle D.A. (2003). Herbivore-mediated linkages between aboveground and belowground communities. Ecology.

[56] de Faccio Carvalho P.C., Anghinoni I., de Moraes A., de Souza E.D., Sulc R.M., Lang C.R., Flores J.P., Lopes M.L., da Silva J.L., Conte O. (2010). Managing grazing animals to achieve nutrient cycling and soil improvement in no-till integrated systems. Nutr. Cycl. Agroecosyst..

[57] Anderson T.-H., Domsch K.H. (1989). Ratios of microbial biomass carbon to total organic carbon in arable soils. Soil Biol. Biochem..

[58] Lu J., SantoDomingo J.W., Hill S., Edge T.A. (2009). Microbial Diversity and Host-Specific Sequences of Canada Goose Feces. Appl. Environ. Microbiol..

[59] Baron S. (1996). Medical Microbiology.

[60] Mor G., Kwon J.-Y. (2015). Trophoblast-microbiome interaction: A new paradigm on immune regulation. Am. J. Obstet. Gynecol..

[61] Parat C., Chaussod R., Lévéque J., Dousset S., Andreux F. (2002). The relationship between cupper accumulated in vineyard calcareous soil and soil organic matter and iron. Eu. J. Soil Sci..

[62] Viti C., Quaranta D., De Philippis R., Corti G., Agnelli A., Cuniglio R., Giovannetti L. (2008). Characterizing cultivable soil microbial communities from copper fungicide-amended olive orchard and vineyard soils. World J. Microbiol. Biotechnol..

[63] Duplay J., Semhi K., Errais E., Imfeld G., Babcsanyi I., Perrone T. (2014). Copper, zinc, lead and cadmium bioavailability and retention in vineyard soils (Rouffach, France): The impact of cultural practices. Geoderma.

[64] Besnard E., Chenu C., Robert M. (2000). Influence of organic amendments on copper distribution among particle-size and density fractions in Champagne vineyard soils. Environ. Pollut..

[65] Lucia M., André J.M., Bernadet M.D., Gontier K., Gérard G., Davail S. (2007). Concentrations of metals (zinc, copper, cadmium, and mercury) in three domestic ducks in France: Pekin, muscovy, and mule ducks. J. Agric. Food Chem..

[66] Chiou P.W.S., Chen K.L., Yu B. (1997). Toxicity, tissue accumulation and residue in egg and excreta of copper in laying hens. Anim. Feed Sci. Technol..

[67] Skřivan M., Skřivanová V., Marounek M. (2006). Effect of various copper supplements to feed of laying hens on Cu content in eggs, liver, excreta, soil, and herbage. Arch. Environ. Contam. Toxicol..

[68] Bortey-Sam N., Nakayama S.M., Ikenaka Y., Akoto O., Baidoo E., Yohannes Y.B., Mizukawa H., Ishizuka M. (2015). Human health risks from metals and metalloid via consumption of food animals near gold mines in Tarkwa, Ghana: Estimation of the daily intakes and target hazard quotients (THQs). Ecotoxicol. Environ. Saf..

[69] Falandysz J. (1991). Manganese, copper, zinc, iron, cadmium, mercury and lead in muscle meat, liver and kidneys of poultry, rabbit and sheep slaughtered in the northern part of Poland, 1987. Food Addit. Contam..

