# Supplementary material for: Geese Reared in Vineyard: Soil, Grass and Animals Interaction
Source: Animals (Basel). 2019 Apr 19;9(4):179. doi: 10.3390/ani9040179 (PMC6523708; doi:10.3390/ani9040179)
Supplement: Supplementary file 1 [file animals-09-00179-s001.pdf]

Table S1. Morphological description by Schoeneberger *et al*. (2012) of the profiles of the vineyard soils with a High (HGD) and Low (LGD) Geese Density, and of the soil of the control vineyard Without Geese (WG), Cannara (PG, central Italy).

Landform, hill; Exposure, S–SE; Slope, 5%; Altitude, 250 m a.s.l Mean annual air temperature: 14.8°C; Mean annual precipitation, 79.4 mm; Parent material, fluvial and lacustrine sediments; Soil classification, mixed, calcareous, mesic Typic Haplustept (Soil Survey Staff, 2014).

Soil use	Horizons	Depth /cm	Colour[a]	Texture[b]	Skeleton[c] /%	Structure[d]	Consistency[e]	Plasticity[f]	Roots[g]	Boundary[h]	Other observations
WG	Ap1	0–7	2.5YR5/4	sic	<1	2fm sbk	ns	p	3vf,f	cw	Abundant $CaCO_3$ concretions.
	Ap2	7–21	2.5YR5/4	sac	<1	3fm abk	ns	sp	3vf,f	cs	Common $CaCO_3$ concretions.
	Bw	21–36	2.5YR5/4.	sic	2-3	3fm abk	s	p	2vf,f, 1m	cs	Common $CaCO_3$ and few Mn concretions.
	BC	36–54	2.5YR5/6	sic	<1	1fm sbk	ns	p	1vf	cw	Abundant $CaCO_3$ and few Mn concretions.
	C	54–64	2.5YR5/4	c	<1	1fm sbk	-	-	2vf,f, 1m	cw	Common $CaCO_3$ and few Mn concretions.
	Cg	64–74+	2.5YR6/4	sic	<1	1 sbk	-	-	2f	-	Common $CaCO_3$ and few Mn concretions.
LGD	Ap1	0–10	10YR4/3	sic	<1	3fm sbk	ss	sp	3m	cw	-
	Ap2	10–20	7.5YR4/2	c	<1	2f sbk	ss	p	1vf,f,m	cw	-
	Bw	20–27	10YR5/3	c	<1	2m abk	s	p	2vf,f	cw	-

	BC1	27-44	10YR5/6	c	<1	2m abk	vs	vp	2vf,f; 1m	cs	Few Mn concretions and reddish streaks (7.5 YR5/6)
	BC2	44-54	10YR6/4	sac	-	2m abk	s	p	2f,m; 1co	cs	Few Mn concretions and reddish streaks (7.5 YR5/6)
	C	54-69+	10YR 5/4	sic	-	1m sbk	vs	vp	1 vf,f,m,co	-	-
HGD	Ap1	0–6	10YR 4/3	cl	<1	2f sbk	ss	p	1vf,f	cs	-
	Ap2	6–15	10YR 4/2	cl	<1	2fm sbk	ss	p	1f 1co	cs	-
	Bw1	15–29	10YR 5/4	cl	<1	2fm sbk	ss	p	2 vf,f,m	cs	-
	Bw2	29–41	10YR 5/4	c	<1	2m sbk	vs	vp	1 vf,f	cs	-
	Bw3	41–54	10YR 5/4	c	1	1m sbk	vs	vp	3vf,f; 2m	cs	-
	C	54–70+	10YR 5/4	c	90	1f sbk	-	-	2v_1 vf	-	-

[a] moist and crushed, according to the Munsell Soil Colour Charts.
[b] sic, silty clay; sac, sandy clay; cl, clay loam; c, clay.
[c] volume percent of particles > 2 mm.
[d] 1 = weak, 2 = moderate, 3 = strong; f = fine, m = medium, c = coarse; cr = crumb, abk = angular blocky, sbk = subangular blocky.
[e] m=moist, fr=friable, fi=firm; w=wet, ns=not sticky, ss=slightly sticky, s=sticky, vs=very sticky.
[f] w=wet, np=not plastic, sp=slightly plastic, p=plastic, vp=very pastic, .
[g] 0 = absent, v_1 = very few, 1 = few, 2 = plentiful, 3 = abundant; vf = very fine, f = fine, m = medium, co = coarse.
[h] c = clear; w = wavy, s = smooth.
